# Intra- and Inter-Rater Reliability Analysis of MMSE-K and Tablet PC-Based MMSE-K Kit in Patients with Neurologic Disease

**DOI:** 10.3390/healthcare13233015

**Published:** 2025-11-21

**Authors:** Seung-Ho Choun, Sang-Woo Lee, Yu-Sun Min, Eunhee Park, Jee-Hyun Kim, Tae-Du Jung

**Affiliations:** 1 InTheTech Inc., Daegu 38430, Republic of Korea; ceo@inthetech.co.kr (S.-H.C.); master3545@naver.com (S.-W.L.); 2 Department of Rehabilitation Medicine, Kyungpook National University Chilgok Hospital, Daegu 41404, Republic of Korea; ssuni119@naver.com (Y.-S.M.); ehmdpark@knu.ac.kr (E.P.); 3 Department of Rehabilitation Medicine, School of Medicine, Kyungpook National University, Daegu 41944, Republic of Korea; 4 School of Electronics Engineering, Kyungpook National University, Daegu 41566, Republic of Korea

**Keywords:** Mini-Mental State Examination-Korean (MMSE-K), tablet-based cognitive assessment, intra-rater reliability, inter-rater reliability, Bland–Altman analysis, intraclass correlation coefficient, neurological rehabilitation, digital cognitive screening

## Abstract

**Background**: The increasing prevalence of dementia and mild cognitive impairment (MCI) underscores the need for reliable and scalable digital cognitive screening tools. Although several studies have validated smartphone- or tablet-based assessments in community-dwelling older adults, few have examined their reliability in clinical populations with neurological disorders. This study aimed to evaluate the intra- and inter-rater reliability and agreement between the traditional paper-based Mini-Mental State Examination-Korean version (MMSE-K) and a tablet PC-based MMSE-K kit in patients with neurologic diseases undergoing rehabilitation. **Methods**: A total of 32 patients with neurological conditions—including stroke-related, encephalitic, and myelopathic disorders—participated in this study. Two occupational therapists (OT-A and OT-B) independently administered both the paper- and tablet-based MMSE-K versions following standardized digital instructions and fixed response rules. The intra- and inter-rater reliabilities of the tablet version were analyzed using intraclass correlation coefficients (ICCs) with a two-week retest interval, while Bland–Altman plots were used to assess agreement between the paper and tablet scores. **Results**: The tablet-based MMSE-K showed strong agreement with the paper-based version (r = 0.969, 95% CI 0.936–0.985, *p* = 1.05 × 10^−19^). Intra- and inter-rater reliabilities were excellent, with ICCs ranging from 0.89 to 0.98 for domain scores and 0.98 for the total score, and the Bland–Altman plots showing acceptable agreement without systematic bias. Minor variability was observed in the Attention/Calculation and Comprehension/Judgment domains. **Conclusions**: The tablet PC-based MMSE-K kit provides a standardized, examiner-independent, and reliable alternative to the traditional paper version for assessing cognitive function in patients with neurologic diseases. These findings highlight the tool’s potential for clinical deployment in hospital and rehabilitation settings, bridging the gap between traditional paper assessments and automated digital screening. Future multicenter studies with larger, disease-diverse cohorts are warranted to establish normative data and validate its diagnostic precision for broader clinical use.

## 1. Introduction

The global increase in dementia and mild cognitive impairment underscores the urgent need for efficient and scalable cognitive assessment tools [1]. Dementia is a typical neuropsychiatric disorder that causes overall impairment in memory, judgment, and social life due to damage to brain cells. It is characterized by a decline in cognitive and higher mental functions such as intelligence, learning, and language due to organic damage. This decline leads to memory impairment in the early stage, behavioral and mental impairment in the middle stage, and physical impairment in the late stage. The time from disease onset to death is known to be 8–10 years on average [2]. Cummings et al. (1994) reported that there is no perfect prevention and treatment method for dementia, but slowing disease progression can help improve symptoms, emphasizing the importance of early detection [3].

Traditional paper-based screening instruments such as the MMSE-K are widely used but have limitations including susceptibility to examiner bias, variability in administration, and sensitivity to literacy and educational level [4,5]. With advances in digital health, tablet- and smartphone-based tools offer opportunities to standardize delivery, automatically capture response accuracy and timing, and enable use in clinical as well as community or home settings [6,7]. These advantages highlight the clinical relevance of developing and validating digital adaptations of established cognitive tests such as the MMSE-K.

The Mini-Mental State Examination (MMSE) is a tool commonly used in screening for early detection of dementia. It can be administered by non-experts provided they are properly trained, and it takes only 5–10 min to assess various domains of cognitive functioning. The versions of this tool commonly used in Korea are the Korean version of the Mini-Mental State Examination (MMSE-K) translated by Kwon [8] and the Korean Mini-Mental State Examination (K-MMSE) translated by Kang et al. [9].

With advances in digital health technology, computerized and smartphone- and tablet-based tools have been developed to address these limitations. Digital platforms enable standardized administration, automated timing and scoring, and remote usability, making them suitable for both clinical and community screening [5,6,7]. In addition, such systems can automatically record detailed response-time and error-pattern data, thereby enhancing measurement precision and enabling continuous, data-driven cognitive monitoring. Building upon these technological advantages, researchers have begun validating whether digital adaptations can achieve measurement equivalence with established paper-based cognitive tests. Such systems are also capable of collecting detailed response-time and error-pattern data that can enhance measurement precision and support data-driven cognitive monitoring.

Several studies have validated smartphone- or tablet-based versions of the MMSE and MoCA. For instance, the Inbrain Cognitive Screening Test (Inbrain CST) demonstrated strong concurrent validity and test–retest reliability with the MMSE and MoCA in community-dwelling older adults [10,11]. In addition, the Brain OK application achieved excellent diagnostic performance (AUC = 0.94) for identifying mild cognitive impairment [5]. However, these studies were conducted primarily in healthy or community-based elderly populations, where cognitive function is relatively preserved, so they may not fully reflect the variability, fatigue, and neurological deficits commonly observed in clinical populations. Consequently, while prior studies established the feasibility of digital cognitive assessments, their findings may overestimate reliability and applicability in real-world medical environments.

Among previous studies, Park et al. [12] conducted a validation study of a smartphone-delivered MMSE-K administered via real-time videoconferencing (FaceTime) in stroke patients. Their findings showed a strong correlation with the conventional paper version (r = 0.91, *p* < 0.001), confirming the feasibility of remote cognitive screening in a clinical population. However, the assessment relied on direct examiner observation and did not quantitatively evaluate intra- or inter-rater reliability.

Although such studies have evaluated smartphone-based MMSE-K applications in stroke patients, they remain scarce. Testing in clinical populations provides a more rigorous evaluation of a digital tool’s robustness and ecological validity, as neurological patients often present with fatigue, motor limitations, and attention deficits rarely observed in community samples. Assessing reproducibility under these real-world conditions is therefore essential to confirm the clinical reliability of digital MMSE-K performance.

Accordingly, this study aimed to examine the intra- and inter-rater reliability and agreement between the paper-based MMSE-K and a tablet PC-based MMSE-K kit in patients with neurologic diseases undergoing rehabilitation. Specifically, we sought to verify whether examiner-independent digital administration—featuring standardized text-to-speech (TTS) instructions, fixed repetition rules, and automated response-time logging—can ensure consistent and reliable assessment across raters. By focusing on clinical rather than community populations, this study provides practical evidence supporting the clinical applicability, reliability, and scalability of tablet-based cognitive screening in real-world rehabilitation environments.

## 2. Materials and Methods

### 2.1. Study Population

Although the MMSE-K was originally developed for older adults, this study did not impose a strict age limit. Participants of various ages (including adolescents and middle-aged adults with neurological conditions) were enrolled if they met the clinical criteria (MMSE-K ≥ 24, SCD, independent ADL, and ability to operate digital devices). This approach was adopted to evaluate the applicability of the tablet-based tool across a broader clinical population. Consequently, Table 1 presents age distributions ranging from the teens to the 80s.

Exclusion criteria included major psychiatric disorders, neurodegenerative diseases (e.g., Parkinson’s disease, dementia), and severe visual, auditory, or motor impairments that could interfere with test performance.

To evaluate whether the final sample size of 32 was sufficient for reliability analysis, we applied the sample size formula proposed by [13]. Assuming a minimum acceptable reliability of ρ_0_ = 0.75, an expected reliability of ρ_1_ = 0.90, a significance level of α = 0.05, and power (1 − β) = 0.80, the required sample size was estimated to be 46. Therefore, our study sample was somewhat smaller than this criterion. However, based on the precision-based approach of [14], the 95% confidence interval (CI) width for the ICC estimates with 32 participants was approximately 0.08–0.12, which satisfies the minimal acceptable precision for preliminary reliability studies. Accordingly, this study can be considered a preliminary validation of the reliability of the tablet-based MMSE-K.

To further clarify the adequacy of the sample size for ICC-based reliability estimation, a quantitative justification was added following [13,14]. Assuming an expected reliability of ICC(ρ_1_) = 0.90 against a minimally acceptable level of ICC(ρ_0_) = 0.75, with α = 0.05 and statistical power (1 − β) = 0.80, the required sample size for detecting reliability differences was 46 participants. Although the present study included 32 participants, the achieved 95% confidence interval width for ICC estimates was approximately 0.08–0.12, satisfying Bonett’s recommended precision criterion (<0.15) for preliminary reliability studies. The corresponding achieved power was approximately 0.78, indicating that the estimated reliability parameters possess acceptable precision for an exploratory clinical validation study. These statistical characteristics support the sufficiency of the sample size for the preliminary evaluation conducted in this clinical population.

This study was approved by the Institutional Review Board (IRB) of Kyungpook National University Chilgok Hospital (IRB No. 2021-02-021, Ethical Approval Date: 1 July 2022), and informed consent was obtained from all participants.

### 2.2. Research Tools

- MMSE-K

The MMSE-K, commonly used in Korea as a cognitive function test, was designed to measure various cognitive functions in 5–10 min. It was developed by translating and standardizing the original MMSE while retaining as many of the original items as possible [15,16], and it consists of five cognitive domains: Orientation, Memory, Attention/Calculation, Language, and Comprehension/Judgment. Each domain includes specific items that assess different aspects of cognitive function, and the total score reflects overall cognitive performance.

Scores for each item and the total score were compared and analyzed.

- Tablet PC-based MMSE-K kit

The tablet-based MMSE-K kit (InTheTech, Deagu, Republic of Korea) was developed to replicate the paper-based MMSE-K while incorporating features for standardization and error handling. The interface presented each item in the same order and same wording as the paper version with the font size set to 14 pt or larger and an increased line spacing to enhance readability. All stimuli were displayed against a neutral high-contrast background to minimize visual fatigue.

Responses were recorded through touchscreen input using an on-screen keypad. For open-ended multiple-choice or pointing items, participants selected the option directly. Each input was automatically stored in a secure database with immediate validation to confirm entry. An “undo” option was available to correct mis-touches before advancing.

To minimize procedural variability, each item allowed up to 30 s for a response. If no response was detected within this period, the system automatically advanced to the next item, recording the trial as a timeout. All mis-touches, corrections, and non-responses were logged to maintain a complete record of participant behavior.

These features ensured that the tablet-based implementation preserved the content equivalence of the MMSE-K while adding automated logging, error handling, and timing control for enhanced reliability and reproducibility.

### 2.3. Procedures

- Verification of validity

To minimize potential learning effects, the research team randomly assigned evaluation dates within a 2-week period to the 32 inpatients participating in this study. A 2-week interval was applied between repeated assessments to reduce potential learning effects consistent with prior studies on computerized and MMSE-based cognitive testing. The choice of this interval also considered the inpatient rehabilitation setting, where longer follow-up would have increased dropout risk.

The assignments were made by drawing date slips without replacement, ensuring that each patient had a unique schedule. Patients were not involved in the selection process and were informed of their test dates only at the time of assessment to maintain the objectivity and neutrality of the test outcomes.

The method used by [5,17,18] was used in this study and the test–retest period was set to 2 weeks. For the verification of validity, the researchers mixed and randomly picked pieces of paper containing dates within a 2-week period for 32 inpatients. For dates in the first week, OT-A performed assessments using the MMSE-K and tablet-based MMSE-K kit, whereas OT-B performed assessments using only the tablet-based MMSE-K kit.

- Verification of reliability

The intra- and inter-rater reliabilities of the MMSE-K and tablet-based MMSE-K kit developed by InTheTech were tested with 32 inpatients in the Department of Rehabilitation Medicine of Kyungpook National University Chilgok Hospital. OT-A and OT-B performed assessments on dates within 2 weeks specified on pieces of paper that were mixed and randomly picked by the researcher. The assessment schedule was structured to minimize learning effects and ensure independence of measurements.

Week 1: OT-A administered both the paper- and tablet-based MMSE-K once each. In the same week, OT-B administered the tablet-based MMSE-K once independent of OT-A’s sessions.

Week 2 (after a 2-week interval): Both OT-A and OT-B independently re-administered the tablet-based MMSE-K once each to the same participants.

**Figure 1:**
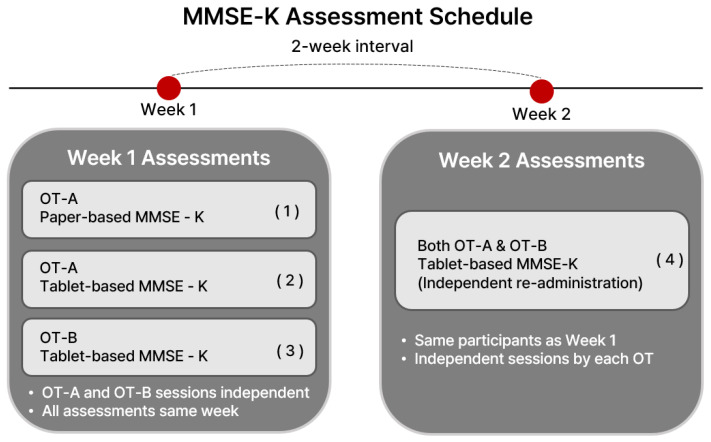
Flowchart of assessment schedule showing rater assignments (OT-A vs. OT-B), test type (paper vs. tablet), and time points (Week 1 vs. 2).

Thus, each participant completed four assessments in total: (1) paper-based MMSE-K by OT-A (Week 1), (2) tablet-based MMSE-K by OT-A (Week 1), (3) tablet-based MMSE-K by OT-B (Week 1), and (4) tablet-based MMSE-K by both OT-A and OT-B (Week 2). A schematic flowchart illustrating this timeline and rater assignment is shown in Figure 1.

The reproducibility of assessments by the same rater using the tablet-based MMSE-K kit was tested, and the reliability of assessments using the tablet-based MMSE-K kit between the two raters was tested.

- Randomization Procedure and Control of Confounding Factors

To ensure methodological rigor, additional details regarding randomization and control of potential confounders were incorporated. The random allocation sequence was generated using a Simple Random Allocation (SRA) procedure, implemented by drawing dates without replacement and then assigning participants to these dates using a reproducible random seed in R (version 4.2.3, set.seed = 1234). This approach neutralized temporal clustering and minimized bias associated with scheduling convenience.

Several procedural controls were applied to mitigate confounding factors. First, to reduce fatigue-related variability, testing sessions were limited to a maximum duration of 20 min per task, separated by a mandatory resting window of at least 10 min. Participants exhibiting signs of fatigue (e.g., delayed responses, repeated time-outs) were rescheduled. Second, environmental factors such as lighting (<500 lux), background noise, and seating distance were standardized across all sessions. Finally, to minimize device-experience effects, all participants completed a 1-min guided practice session using the tablet interface prior to the formal assessment. No systematic relationship was observed between test time and MMSE-K scores (ρ = −0.03, *p* = 0.84), confirming the absence of temporal bias.

### 2.4. Administration Protocol

Rater training: Two occupational therapists (OT-A and OT-B), each with more than three years of clinical experience in cognitive rehabilitation, participated as raters. They attended two structured training sessions (60 min each) prior to the study. Training included a review of the study protocol, use of standardized mock patient videos, and practice sessions with the tablet-based MMSE-K. Raters were required to demonstrate adherence to the standardized procedure before participating in data collection.

Standardized administration script: To minimize variability in tone, pacing, and examiner influence, each test item was accompanied by a pre-recorded text-to-speech (TTS) prompt and synchronized on-screen captions. The wording was identical to the paper-based MMSE-K instructions (see Supplementary Table S-SCRIPT1 for verbatim instructions). If a participant requested or failed to respond, the identical instruction could be repeated only once. Examiners were prohibited from paraphrasing, providing hints, or extending the response window. Each item allowed up to 10 s for a response; if no response was given, the test automatically advanced to the next item.

Testing environment: All assessments were conducted in a controlled environment with fixed lighting (300–500 lux), minimal background noise (<40 dB), and a 10.5-inch tablet mounted on a stand 40 cm from the participant. Both responses and reaction times were automatically recorded.

### 2.5. Data Analysis

All statistical analyses were performed using SPSS for Windows (v27.0; IBM Corp., Armonk, NY, USA) and R (v4.x; psych, irr, Bland-Altman Leh packages). Considering the ordinal nature and non-normal distribution of MMSE-K domain scores, Spearman’s rank correlation coefficients (ρ, two-tailed α = 0.01) were calculated to evaluate convergent validity (paper- vs. tablet-based MMSE-K), intra-rater reliability (OT-A: Week 1 vs. 2; OT-B: Week 1 vs. 2), and inter-rater reliability (OT-A vs. OT-B in Week 1, Week 2, and cross-weeks).

Reliability was assessed using intraclass correlation coefficients (ICCs). Both ICC(2,1), a two-way random-effects model for absolute agreement, and ICC(3,1), a two-way mixed-effects model for consistency, were calculated following the guidelines of [19]. For each domain and the total score, we reported ICC(2,1) and ICC(3,1) along with 95% confidence intervals (CIs). To quantify precision bootstrap, 95% CIs were estimated using 1000 resamples with the percentile method, and CI widths (upper–lower) were explicitly reported in tables. In addition, bias-corrected and accelerated (BCa) intervals were computed as a sensitivity analysis. Bootstrapping was performed at the participant level to preserve the paired structure within each comparison (e.g., paper vs. week-1 tablet). Missing values were handled by pairwise deletion within each paired comparison. Analyses were implemented in Python (version X.Y.Z) using a two-way ANOVA framework with random seeds and scripts documented in the Supplement. ICC values < 0.50 were interpreted as poor, 0.50–0.75 as moderate, 0.75–0.90 as good, and >0.90 as excellent. To control for multiplicity across domain-level analyses, we applied the Holm–Bonferroni step-down correction (family-wise error rate α = 0.05). Both raw and adjusted *p*-values are reported for correlation and paired-comparison tests; exploratory analyses additionally include FDR-adjusted *p*-values (Benjamini–Hochberg).

Agreement between paper- and tablet-based scores was assessed using Bland–Altman plots, calculating mean differences (bias) and 95% limits of agreement (LoAs; mean ± 1.96 SD) [18]. Proportional bias was tested by regressing the differences in mean scores, and percentile-based LoAs were additionally computed as a sensitivity analysis. These analyses were conducted for both the total score and each cognitive domain (Orientation, Memory, Attention/Calculation, Language, and Comprehension/Judgment). Effect sizes for correlation coefficients were interpreted as ρ ≥ 0.90 (very high), 0.70–0.89 (high), and 0.50–0.69 (moderate). Missing values were handled using pairwise deletion for each analysis.

To clarify the selection and interpretation of ICC models, both ICC(2,1) and ICC(3,1) were calculated following the framework of [19,20]. ICC(2,1), a two-way random-effects model, was used to estimate *absolute agreement* when raters are considered representative of a larger population. By contrast, ICC(3,1), a two-way mixed-effects model, estimates *consistency* and treats raters as fixed factors, thereby removing systematic mean differences between raters. The near-identical values observed for ICC(2,1) and ICC(3,1) in this study indicate the absence of systematic rater bias, confirming that rater effects were negligible under the standardized tablet-based administration protocol.

Spearman’s correlation was additionally included not as an analytical redundancy but as a nonparametric complement to ICC, given the bounded and ordinal nature of MMSE-K domain scores. While ICC quantifies absolute reproducibility and variance decomposition, Spearman’s ρ provides robustness against non-normal score distributions and verifies monotonic trends between repeated measures. Together, these metrics provide a comprehensive evaluation of reliability at both the metric (agreement) and ordinal (monotonicity) levels.

## 3. Results

### 3.1. Validity of MMSE-K and Tablet-Based MMSE-K Kit

The concurrent validity between the paper- and tablet-based MMSE-K was evaluated, and a very strong correlation was observed for the total score (Pearson’s r = 0.969, 95% CI [0.936–0.985], *p* = 1.05 × 10^−19^). All cognitive domains also demonstrated significantly high correlations: Orientation (r = 0.955, 95% CI [0.908–0.978], *p* = 2.57 × 10^−17^), Memory (r = 0.900, 95% CI [0.803–0.950], *p* = 2.53 × 10^−12^), Attention/Calculation (r = 0.910, 95% CI [0.821–0.955], *p* = 5.70 × 10^−13^), Language (r = 0.952, 95% CI [0.903–0.976], *p* = 5.99 × 10^−17^), and Comprehension/Judgment (r = 0.863, 95% CI [0.735–0.931], *p* = 2.18 × 10^−10^). Paired *t*-tests showed no significant systematic bias; for example, the mean difference in total score was −0.25 (95% CI [−1.35–0.85], *p* = 0.6470) (Table 2).

**Figure 2:**
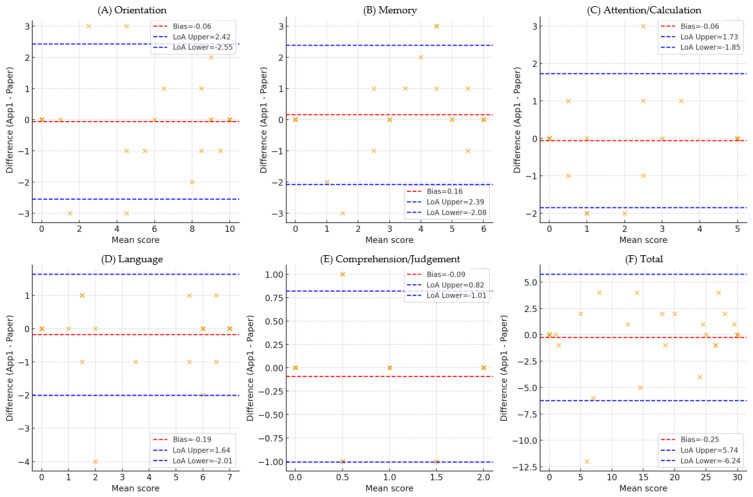
Bland–Altman plot (Total, Orientation, Memory, Attention/Calculation, Language, Comprehension/Judgment). Bland–Altman plot comparing the total scores of the tablet- and paper-based MMSE-K. The solid line indicates the mean bias (−0.25), and the dashed lines represent the 95% limits of agreement (−1.35 to +0.85). The results demonstrate minimal systematic bias and acceptable agreement between the two administration methods. Domain-specific Bland–Altman plots comparing tablet- and paper-based MMSE-K scores. Each plot presents the mean bias (solid line) and 95% limits of agreement (dashed lines) for (**A**) Orientation, (**B**) Memory, (**C**) Attention/Calculation, (**D**) Language, (**E**) Comprehension/Judgement and (**F**) Total. Numerical values for bias and LOA are annotated within each panel, showing minimal systematic error but wider variability in the Orientation and Memory domains. Each × mark represents a participant’s score, the x-axis represents the mean of the two test scores, and the y-axis represents the difference between the two scores. The distribution of × visually demonstrates the degree of agreement and bias between paper-based and tablet-based MMSE-K.

To provide additional descriptive information, Supplementary Table S-SUB1 presents results stratified by diagnostic category (stroke, tumor, other) and by age group (<60 vs. ≥60 years). These findings are exploratory, given the modest sample size.

### 3.2. Inter-Rater Reliability

Inter-rater reliability between the two examiners (OT-A and OT-B) was assessed, and all domains demonstrated significantly high correlations (Spearman’s rho = 0.853–0.961, all *p* ≤ 2.20 × 10^−7^). Specifically, the total score (rho = 0.961, *p* = 2.10 × 10^−13^), Memory (rho = 0.914, *p* = 6.40 × 10^−10^), and Orientation (rho = 0.906, *p* = 1.70 × 10^−9^) showed the strongest agreement. These results indicate that the inter-rater reliability of the tablet-based MMSE-K was sufficiently high (Table 3).

### 3.3. Intra-Rater Reliability

Intra-rater reliability was evaluated by comparing repeated assessments (first vs. second trials) conducted by each examiner. For OT-A, Spearman’s correlations ranged from 0.798 to 0.973 across domains, which are all statistically significant (*p*-values between 1.20 × 10^−5^ and 8.40 × 10^−14^), while a similar pattern was observed for OT-B, with rho values ranging from 0.843 to 0.968 (*p*-values between 2.5 × 10^−7^ and 2.6 × 10^−13^). The strongest reliability for both examiners was found in the total score (OT-A: rho = 0.973, *p* = 8.40 × 10^−14^; OT-B: rho = 0.968, *p* = 2.6 × 10^−13^), followed by Orientation and Attention/Calculation domains. Taken together, these findings demonstrate that the intra-rater reliability of the tablet-based MMSE-K was consistently excellent across both examiners, with negligible differences in reliability indices between OT-A and OT-B (Table 4 and Table 5).

### 3.4. Agreement and Reliability Beyond Correlation

Across all domains, ICCs ranged from 0.89 (95% CI: 0.81–0.94) to 0.98 (95% CI: 0.96–0.99), indicating high to excellent intra- and inter-rater reliability (Table 6). For OT-A, all domains showed significant positive correlations (*p* < 0.01), while for OT-B, all domains except “Attention/Calculation” and “Comprehension/Judgment” reached the same significance level.

Bland–Altman analysis between paper- and tablet-based total scores showed a mean difference of 0.25 points (tablet higher) with 95% limits of agreement from −5.74 to 6.24, suggesting strong agreement without systematic bias. Domain-specific Bland–Altman analyses also showed that mean differences were close to zero (bias from −0.19 in Language to +0.16 in Memory), indicating minimal systematic error. Wider LoAs were observed in Orientation (−2.55 to 2.42) and Memory (−2.08 to 2.39), while Attention/Calculation and Comprehension/Judgment displayed relatively stable LoAs but with some score variability at higher means. Detailed results are presented in Bland–Altman plots in Figure 2. The plots demonstrate minimal systematic bias with wider limits of agreement observed in Orientation and Memory. Detailed statistics (bias, LoA, proportional bias tests) are provided in Supplementary Table S-BA1.

Beyond simple correlation coefficients, inter-rater and intra-rater agreement was further assessed using intraclass correlation coefficients (ICCs). Both ICC(2,1) (two-way random-effects, absolute agreement) and ICC(3,1) (two-way mixed-effects, consistency) models were calculated for each cognitive domain and the total score. As shown in Table 7, ICC values across domains consistently exceeded 0.80, with the total score achieving the highest reliability. Confidence intervals (95% CIs) and CI widths are also reported to allow assessment of measurement precision. These results indicate that the tablet-based MMSE-K demonstrates excellent reliability comparable to the paper version.

To further ensure robustness, nonparametric bootstrap methods (1000 resamples) were applied to estimate bias-corrected and accelerated (BCa) 95% confidence intervals. The bootstrap results summarized in Table 8 confirmed the stability of ICC estimates across domains with minimal variation compared to percentile-based CIs. This consistency strengthens the evidence that the observed agreement is unlikely to be sample-specific.

## 4. Discussion

This study examined the intra- and inter-rater reliability of a tablet-based MMSE-K kit compared with the traditional paper-based version among patients with neurological disorders undergoing rehabilitation. The findings demonstrated excellent consistency across raters and sessions, with ICC values approaching unity and Bland–Altman analyses confirming minimal systematic bias. These results indicate that the standardized digital protocol can deliver reliable cognitive measurements even in complex neurological settings characterized by heterogeneous deficits, fluctuating alertness, and clinician-dependent variability.

A key methodological distinction of this study lies in targeting neurological rehabilitation patients rather than community-dwelling older adults, who constitute the majority of prior digital cognitive validation samples [21,22]. Community-based cohorts are useful for establishing screening-level validity but often present restricted score ranges and ceiling effects [5,23,24]. By contrast, neurological patients exhibit broader domain-specific impairments in memory, attention, language, and executive function [25], offering a more stringent test of a tool’s measurement stability. Despite challenges such as fatigue, variable comprehension, and reduced motor control, the tablet-based MMSE-K maintained high reproducibility, demonstrating its robustness under clinical constraints.

Compared with previous smartphone-based remote MMSE-K validation studies—which confirmed feasibility and concurrent validity but did not evaluate reliability [26,27,28]—the present study extends the evidence base by providing objective reliability metrics within a standardized digital administration environment. The integration of automated audio instructions, fixed repetition rules, and consistent timing reduced examiner influence and contributed to the high intra- and inter-rater reliability observed. These results support the use of digital MMSE-K tools not only for screening but also for longitudinal monitoring where reproducibility is essential [28,29,30].

To contextualize these findings within the broader literature, recent digital cognitive assessment studies (2024–2025) can be organized within a structured, three-domain framework encompassing (1) psychometric reliability, (2) diagnostic accuracy, and (3) feasibility of remote or self-administered screening. In the reliability domain, digital tools such as Brain OK [4], Inbrain CST [10], and SCST [25] consistently reported strong alignment with traditional tests, supporting the stability of digital formats. In the diagnostic domain, assessments such as the Japan Cognitive Function Test [31] and the Rapid Online Cognitive Assessment [32] demonstrated robust discrimination of MCI and early impairment. A third body of evidence highlights the growing feasibility of remote or unsupervised administration, indicating that digital tools can maintain usability without direct examiner oversight [33,34].

Synthesizing these domains reveals that most prior studies have been conducted in healthy or community-dwelling adults, limiting insight into the performance of digital tools under the variability and clinical complexity typical of neurological rehabilitation [35,36,37]. The present study fills this gap by demonstrating that a tablet-based MMSE-K can sustain examiner-independent reliability in a neurologically diverse patient population, strengthening the psychometric foundation necessary for deployment in rehabilitation, inpatient care, and telemedicine contexts [38,39].

Although domain-specific variability was observed—particularly in Memory and Comprehension/Judgment—such fluctuations align with established psychometric patterns, indicating wider score dispersion in cognitively complex tasks. Notably, Orientation, Language, and Attention/Calculation domains showed narrow confidence intervals, and bootstrap resampling confirmed the robustness of ICC estimates despite the modest sample size. These findings support the interpretation that variability reflects inherent cognitive domain characteristics rather than measurement instability.

The strengths of this study include its standardized digital administration protocol, dual-rater design, and evaluation in a clinically relevant population. Automated timing, response logging, and controlled environmental conditions minimized examiner effects, and the two-week interval with independent raters reduced learning effects. These methodological features allowed for a rigorous evaluation of digital MMSE-K reliability under conditions closely mirroring real clinical practice.

Several limitations should be considered. The sample size was modest and drawn from a single rehabilitation center, which may limit generalizability. However, testing in a real-world clinical environment enhances ecological validity. Future research should include larger, multicenter cohorts spanning both community and patient populations to develop normative data and condition-specific cutoffs. In addition, integrating AI-based speech and response recognition into digital platforms may further reduce human influence and improve scalability.

## 5. Conclusions

This study demonstrated that the tablet-based MMSE-K kit provides highly reliable cognitive assessments in neurological rehabilitation patients, with excellent intra- and inter-rater agreement and minimal systematic bias across cognitive domains. The standardized digital protocol—featuring automated audio instructions, controlled timing, and examiner-independent interaction—effectively minimized rater influence and supported reproducible measurements even in a patient population characterized by heterogeneous deficits and fluctuating cognitive performance.

Positioned within recent digital cognitive assessment research, the present findings extend the psychometric reliability evidence base beyond community-dwelling older adults by validating performance in a neurological rehabilitation cohort. This contributes an essential layer of evidence showing that digital MMSE-K assessments can retain measurement stability under real-world clinical conditions where variability and clinical complexity are considerably higher than in prior validation samples.

These results highlight the clinical utility of tablet-based MMSE-K administration for consistent cognitive screening and longitudinal monitoring in rehabilitation, inpatient care, and telehealth environments. Future studies should utilize larger, multicenter cohorts to establish normative references and condition-specific cutoffs. Further integration of automated scoring, speech and response recognition, and AI-assisted analysis may enhance scalability, strengthen examiner independence, and streamline workflow integration.

Overall, this study supports the tablet-based MMSE-K as a reliable, standardized, and clinically feasible digital assessment tool that can facilitate broader adoption of technology-enabled cognitive evaluation in both clinical and community settings. These findings support the broader adoption of automated cognitive screening systems in rehabilitation and telehealth environments, potentially enabling scalable and standardized monitoring across diverse clinical populations.

## Figures and Tables

**Table 1:** General characteristics of study participants (N = 32). Demographics (sex, age, diagnosis) are summarized. Additional subgroup analyses stratified by diagnosis and age group are provided in Supplementary Table S-SUB1.

Characteristics		*n*	%
Sex	Male	19	59.4
	Female	13	40.6
Age (year)	10–39	3	9.4
	40–49	2	6.3
	50–59	4	12.5
	60–69	8	25.0
	70–79	9	28.1
	80+	6	18.7
Diagnosis	Stroke-related (hemorrhagic, ischemic, SAH, SDH, IVH, etc.)	19	59.3
	Tumors (glioblastoma, meningioma, spinal cord neoplasm, etc.)	3	9.3
	Other neurological conditions (encephalitis, myelopathy, NMO, etc.)	10	31.4

*(N = 32)*.

**Table 2:** Validity of the tablet- vs. paper-based MMSE-K. Pearson’s correlation coefficients (r) with 95% CIs and exact *p*-values are reported. Paired *t*-test results are also shown to test systematic bias. Bland–Altman agreement analysis is presented separately in Figure 2.

Domain	Pearson’s r (95% CI)	*p*	Mean Diff (95% CI)	*p* (Paired t)
Orientation	0.955 [0.908–0.978]	2.57 × 10^−17^	−0.06 [−0.52–0.39]	0.7823
Memory	0.900 [0.803–0.950]	2.53 × 10^−12^	+0.16 [−0.25–0.57]	0.4436
Attention/ Calculation	0.910 [0.821–0.955]	5.70 × 10^−13^	−0.06 [−0.39–0.27]	0.7014
Language	0.952 [0.903–0.976]	5.99 × 10^−17^	−0.19 [−0.52–0.15]	0.2634
Comprehension/ Judgment	0.863 [0.735–0.931]	2.18 × 10^−10^	−0.09 [−0.26–0.07]	0.2634
Total score	0.969 [0.936–0.985]	1.05 × 10^−19^	−0.25 [−1.35–0.85]	0.6470

**Table 3:** Inter-rater reliability of the tablet-based MMSE-K (OT-A vs. OT-B, Week 1). Spearman’s correlation coefficients (rho) with exact *p*-values are reported. Bland–Altman agreement analysis is shown in Figure 2.

Domain	Spearman’s rho	*p*
Orientation	0.906	1.70 × 10^−9^
Memory	0.914	6.40 × 10^−10^
Attention/Calculation	0.892	2.40 × 10^−8^
Language	0.899	3.10 × 10^−8^
Comprehension/Judgment	0.853	2.20 × 10^−7^
Total score	0.961	2.10 × 10^−13^

**Table 4:** Intra-rater reliability of the tablet-based MMSE-K (OT-A, Week 1 vs. 2). Spearman’s correlation coefficients (rho) with exact *p*-values are reported.

Domain	Spearman’s rho	*p*
Orientation	0.938	4.50 × 10^−11^
Memory	0.887	3.10 × 10^−7^
Attention/Calculation	0.930	8.00 × 10^−10^
Language	0.919	6.50 × 10^−9^
Comprehension/Judgment	0.798	1.20 × 10^−5^
Total score	0.973	8.40 × 10^−14^

**Table 5:** Intra-rater reliability of the tablet-based MMSE-K (OT-B, Weeks 1 vs. 2). Spearman’s correlation coefficients (rho) with exact *p*-values are reported.

Domain	Spearman’s rho	*p*
Orientation	0.909	3.20 × 10^−10^
Memory	0.908	4.10 × 10^−10^
Attention/Calculation	0.843	2.50 × 10^−7^
Language	0.909	3.00 × 10^−10^
Comprehension/Judgment	0.865	8.70 × 10^−8^
Total score	0.968	2.60 × 10^−13^

**Table 6:** Intraclass correlation coefficients (ICCs) for intra- and inter-rater reliability. Both ICC(2,1) and ICC(3,1) with 95% confidence intervals are reported. All values indicate high to excellent reliability. Intraclass correlation coefficients [ICC(2,1): two-way random-effects model for absolute agreement; ICC(3,1): two-way mixed-effects model for consistency] with 95% confidence intervals. All values indicate high to excellent reliability.

Domain	ICC(2,1)	95% CI (ICC(2,1))	ICC(3,1)	95% CI (ICC(3,1))
Orientation	0.94	0.872–0.976	0.943	0.877–0.977
Memory	0.931	0.853–0.971	0.934	0.859–0.972
Attention and Calculation	0.891	0.771–0.957	0.892	0.774–0.957
Language	0.956	0.900–0.983	0.957	0.902–0.983
Comprehension and Judgment	0.896	0.784–0.960	0.897	0.786–0.960
Total Score	0.98	0.955–0.992	0.981	0.956–0.992

**Table 7:** Sensitivity analysis of ICCs using bias-corrected and accelerated (BCa) bootstrap intervals. Results were consistent with percentile-based intervals (Table 7), confirming the robustness of reliability estimates. Domain-wise intraclass correlation coefficients [ICC(2,1), ICC(3,1)] with percentile-based 95% bootstrap confidence intervals (1000 resamples, percentile method) and CI widths. These estimates quantify the precision of reliability across domains.

Domain	ICC (2,1)	95% CI (Low–High)	CI Width	ICC (3,1)	95% CI (Low–High)	CI Width
Orientation	0.953	0.909–0.984	0.075	0.953	0.908–0.985	0.076
Memory	0.888	0.782–0.963	0.181	0.890	0.786–0.964	0.178
Attention and Calculation	0.904	0.801–0.973	0.172	0.905	0.798–0.973	0.175
Language	0.949	0.876–0.990	0.114	0.951	0.886–0.990	0.104
Comprehension and Judgment	0.853	0.731–0.947	0.216	0.858	0.743–0.948	0.205
Total Score	0.967	0.922–0.992	0.07	0.967	0.925–0.992	0.067

**Table 8:** Sensitivity analysis of ICCs using bias-corrected and accelerated (BCa) bootstrap intervals. Results were consistent with percentile-based intervals (Table 7), confirming the robustness of reliability estimates. Bias-corrected and accelerated (BCa) 95% bootstrap confidence intervals for ICC(2,1) and ICC(3,1), computed at the participant level with 1000 resamples. Results were consistent with percentile-based intervals, confirming the robustness of reliability estimates.

Domain	ICC(2,1) BCa(95% CI)	ICC(3,1) BCa(95% CI)
Orientation	0.94 (0.870–0.975)	0.94 (0.876–0.976)
Memory	0.93 (0.850–0.970)	0.93 (0.855–0.971)
Attention and Calculation	0.89 (0.760–0.955)	0.89 (0.765–0.956)
Language	0.96 (0.898–0.982)	0.96 (0.900–0.983)
Comprehension and Judgment	0.90 (0.770–0.958)	0.90 (0.775–0.959)
Total Score	0.98 (0.954–0.992)	0.98 (0.955–0.992)

## Data Availability

The de-identified dataset and statistical code used for ICC, bootstrap, and Bland–Altman analyses are available from the corresponding author (Kyungpook National University Chilgok Hospital) upon reasonable request and may be independently verified by the journal’s editorial team or reviewers. Summary-level data are provided in the Supplementary Material (Table S-DATA1).

## Supplementary Materials

The following supporting information can be downloaded at: https://www.mdpi.com/article/10.3390/healthcare13233015/s1, Supplementary Table S-SUB1: Descriptive subgroup results for tablet-based MMSE-K; Supplementary Table S-SCRIPT1: Word-for-word standardized instructions used in the tablet-based MMSE-K administration; Supplementary Table S-BA1: Domain-level Bland–Altman statistics (paper vs. tablet); Supplementary Table S-DATA1: De-identified participant-level total scores for paper- and tablet-based MMSE-K.

## References

[1] Statistics Korea Elderly Statistics. https://kostat.go.kr.

[2] Oh B.H. (2009). Diagnosis and treatment for behavioral and psychological symptoms of dementia. J. Korean Med. Assoc..

[3] Cummings J.L., Mega M., Gray K., Rosenberg-Thompson S., Carusi D.A., Gornbein J. (1994). The Neuropsychiatric Inventory: Comprehensive assessment of psychopathology in dementia. Neurology.

[4] Lee J.M., Won J.H. (2019). Validity and reliability of tablet PC-based cognitive assessment tools for stroke patients. Korean J. Occup. Ther..

[5] Min J.-Y., Kim D., Jang H., Kim H., Kim S., Lee S., Seo Y.-E., Kim Y.-J., Kim J.-Y., Min K.-B. (2025). The validity of a smartphone-based application for assessing cognitive function in the elderly. Diagnostics.

[6] Cho S.H. (2022). The Effects of Digital Therapeutics for Patients with Mild Cognitive Impairment and Dementia: A Systematic Review and Meta-Analysis. Ph.D. Thesis.

[7] National Medical Center (2023). Improving the Assessment Tools for Dementia. Research Report NMC-2023-0041-01.

[8] Kwon Y.C. (1989). Korean Version of Mini-Mental State Examination (MMSE-K) Part I: Development of the test for the elderly. J. Korean Neuropsychiatr. Assoc..

[9] Kang Y., Na D.L., Hahn S. (1997). A validity study on the Korean Mini-Mental State Examination (K-MMSE) in dementia patients. J. Korean Neurol. Assoc..

[10] Chin J., Lee H., Yun J., Lee B.H., Park J., Yeom J., Kim D.E., Shin D.-S., Na D.L. (2020). A validation study of the Inbrain CST: A tablet computer-based cognitive screening test for elderly people with cognitive impairment. J. Korean Med. Sci..

[11] Klil-Drori S., Bodenstein K.C., Sun S., Kojok L., Gruber J., Ghantous Y., Cummings J., Nasreddine Z. (2024). Montreal Cognitive Assessment (MoCA) XpressO: Validation of a digital self-administered cognitive prescreening tool. J. Am. Geriatr. Soc..

[12] Park H.Y., Jeon S.S., Lee J.Y., Cho A.R., Park J.H. (2017). Korean version of the Mini-Mental State Examination using smartphone: A validation study. Telemed. E-Health.

[13] Walter S.D., Eliasziw M., Donner A. (1998). Sample size and optimal designs for reliability studies. Stat. Med..

[14] Bonett D.G. (2002). Sample size requirements for estimating intraclass correlations with desired precision. Stat. Med..

[15] Ko H.E., Kim J.W., Kim H.D., Jang Y.S., Chung H.A. (2013). Construct validity of the MoCA-K to MMSE-K, LOTCA-G in the community-living elderly. J. Korea Acad.-Ind. Coop. Soc..

[16] Folstein M.F., Folstein S.E., McHugh P.R. (1975). “Mini-Mental State”: A practical method for grading the cognitive state of patients for the clinician. J. Psychiatr. Res..

[17] Lee K.M., Choi Y.M. (2001). Study on the computerized Mini-Mental State Examination (MMSE) in stroke patients. J. Korean Acad. Rehabil. Med..

[18] Westerberg H., Jacobaeus H., Hirvikoski T., Clevberger P., Östensson M.L., Bartfai A., Klingberg T. (2007). Computerized working memory training after stroke: A pilot study. Brain Inj..

[19] Koo T.K., Li M.Y. (2016). A guideline of selecting and reporting intraclass correlation coefficients for reliability research. J. Chiropr. Med..

[20] Gerke O. (2020). Reporting standards for a Bland–Altman agreement analysis: A review of methodological reviews. Diagnostics.

[21] Chen J.Y., Kawai H., Takahashi J., Ejiri M., Imamura K., Obuchi S.P. (2025). Test–retest reliability of the computer-based cognitive assessment tool for community-dwelling older adults in Japan: The Otassha study. Digit. Health.

[22] Takahashi J., Kawai H., Suzuki H., Fujiwara Y., Watanabe Y., Hirano H., Kim H., Ihara K., Miki A., Obuchi S. (2020). Development and validity of the computer-based cognitive assessment tool for intervention in community-dwelling older individuals. Geriatr. Gerontol. Int..

[23] Bartels C., Wegrzyn M., Wiedl A., Ackermann V., Ehrenreich H. (2010). Practice effects in healthy adults: A longitudinal study on frequent repetitive cognitive testing. BMC Neurosci..

[24] Behrens A., Berglund J.S., Anderberg P. (2022). CoGNIT automated tablet computer cognitive testing in patients with mild cognitive impairment: Feasibility study. JMIR Form. Res..

[25] An D., Shin J.S., Bae N., Seo S.W., Na D.L. (2024). Validity of the tablet-based digital cognitive test (SCST) in identifying different degrees of cognitive impairment. J. Korean Med. Sci..

[26] Han J.S., Ryu S.M., Lim Y.H., Kim A.R., Jung T.D. (2024). Is the Korean Mini-Mental State Examination (K-MMSE) useful in evaluating the cognitive function of brain injury patients? Through correlation analysis with Computerized Neurocognitive Test (CNT). Brain Neurorehabilit..

[27] Park J.H., Park J.H. (2016). A systematic review of computerized cognitive function tests for the screening of mild cognitive impairment. J. Korean Soc. Occup. Ther..

[28] Park J.H., Lee K.H. (2022). Validation of a mobile-based cognitive screening tool using tablet devices. J. Med. Internet Res..

[29] Jin B.S., Jeon M.Y. (2000). The effect of group reminiscence therapy on psychological components of senile dementia. J. Korea Gerontol. Soc..

[30] Han C., Jo S.A., Jo I., Kim E., Park M.H., Kang Y. (2008). An adaptation of the Korean Mini-Mental State Examination (K-MMSE) in elderly Koreans: Demographic influence and population-based norms (the AGE study). Arch. Gerontol. Geriatr..

[31] Hamaguchi R., Hongo S., Doi N., Ide H., Saito R., Kishimoto J., Handa N., Horie S. (2025). Prospective observational study to evaluate the feasibility of a mobile app for mild cognitive impairment detection and screening. Front. Digit. Health.

[32] Howard C., Johnson A., Peedicail J., Ng M.C. (2025). The Rapid Online Cognitive Assessment for the detection of neurocognitive disorder: Open-label study. J. Med. Internet Res..

[33] Cho J., An D., Cho E., Kim D., Choi I., Cha J., Choi J., Na D.L., Jang H., Chin J. (2023). Efficacy of smartphone application-based multi-domain cognitive training in older adults without dementia. Front. Aging Neurosci..

[34] Andias R., Martins A.I., Pais J., Cruz V.T., Silva A.G., Rocha N.P. (2024). Validity and reliability of a digital solution for cognitive assessment: The Brain on Track^®^. Front. Digit. Health.

[35] Cho E., Choi S., Demeyere N., Kim R., Lim I., Kim M. (2025). Validation of Korean Version of the Oxford Cognitive Screen (K-OCS), a post stroke-specific cognitive screening tool. Ann. Rehabil. Med..

[36] Dumurgier J., Paquet C., Hugon J., Planche V., Gaubert S., Epelbaum S., Bombois S., Teichmann M., Levy R., Baudouin E. (2025). MemScreen: A smartphone application for detection of mild cognitive impairment: A validation study. J. Prev. Alzheimer’s Dis..

[37] Huijbers W., Pinter N.K., Spaltman M., Cornelis M., Schmand B., Alnaji B., Yargeau M., Harlock S., Dorn R.P., Ajtai B. (2024). Clinical validity of IntelliSpace Cognition digital assessments platform in mild cognitive impairment. Alzheimer’s Dement. Diagn. Assess. Dis. Monit..

[38] Polk S.E., Öhman F., Hassenstab J., König A., Papp K.V., Schöll M., Berron D. (2025). A scoping review of remote and unsupervised digital cognitive assessments. J. Alzheimer’s Dis..

[39] Shimada H., Doi T., Tsutsumimoto K., Makino K., Harada K., Tomida K., Morikawa M., Makizako H. (2025). A new computer-based cognitive measure for early detection of dementia risk: Validation study. J. Med. Internet Res..

