# Supplementary material for: Intra- and Inter-Rater Reliability Analysis of MMSE-K and Tablet PC-Based MMSE-K Kit in Patients with Neurologic Disease
Source: Healthcare (Basel). 2025 Nov 21;13(23):3015. doi: 10.3390/healthcare13233015 (PMC12692307; doi:10.3390/healthcare13233015)
Supplement: Supplementary file 1 [file healthcare-13-03015-s001.zip › Supplementary Table S-BA1.pdf]

Supplementary Material

Table S-BA1. Domain-level Bland–Altman statistics (paper vs. tablet).

Domain	Bias (Mean Difference)	95% LoA (Lower – Upper)	Proportional Bias (p-value)
Total score	-0.25	-6.24 to 5.74	0.41
Orientation	-0.06	-2.55 to 2.42	0.37
Memory	0.16	-2.08 to 2.39	0.29
Attention & Calculation	-0.06	-1.85 to 1.73	0.45
Language	-0.19	-2.01 to 1.64	0.52
Comprehension/Judgment	-0.09	-1.01 to 0.82	0.34
