# Supplementary material for: Intra- and Inter-Rater Reliability Analysis of MMSE-K and Tablet PC-Based MMSE-K Kit in Patients with Neurologic Disease
Source: Healthcare (Basel). 2025 Nov 21;13(23):3015. doi: 10.3390/healthcare13233015 (PMC12692307; doi:10.3390/healthcare13233015)
Supplement: Supplementary file 1 [file healthcare-13-03015-s001.zip › Supplementary Table S-DATA1.pdf]

Supplementary Material

Table S-DATA1. De-identified participant-level total scores for paper- and tablet-based MMSE-K.

ID	Age	Sex	Paper_Total	Tablet_W1_Total	Tablet_W2_Total
P01	33.0	M	30	10	30
P02	88.0	F	0	0	0
P03	76.0	F	0	0	0
P04	69.0	M	1	0	3
P05	77.0	F	0	0	8
P06	67.0	M	6	0	6
P07	74.0	F	0	0	0
P08	66.0	F	0	0	0
P09	50.0	F	30	10	30
P10	64.0	M	30	10	30
P11	75.0	F	25	9	24
P12	53.0	F	21	7	17
P13	73.0	M	4	1	9
P14	72.0	M	26	8	30
P15	60.0	M	0	0	0
P16	50.0	M	22	7	23
P17	12.0	F	29	10	28
P18	72.0	M	12	3	17
P19	79.0	F	26	9	26
P20	68.0	M	10	4	14
P21	41.0	M	16	6	13
P22	77.0	F	18	5	14
P23	74.0	M	19	6	15
P24	78.0	F	0	0	0
P25	74.0	M	30	10	30
P26	75.0	M	30	10	30

P27	56.0	M	29	9	29
P28	73.0	M	13	4	17
P29	54.0	M	0	0	0
P30	72.0	F	0	0	0
P31	77.0	M	25	9	27
P32	71.0	M	1	0	13
