# Supplementary material for: Intra- and Inter-Rater Reliability Analysis of MMSE-K and Tablet PC-Based MMSE-K Kit in Patients with Neurologic Disease
Source: Healthcare (Basel). 2025 Nov 21;13(23):3015. doi: 10.3390/healthcare13233015 (PMC12692307; doi:10.3390/healthcare13233015)
Supplement: Supplementary file 1 [file healthcare-13-03015-s001.zip › Supplementary Table S-SCRIPT1.pdf]

Supplementary Material

Table S-SCRIPT1. Word-for-word standardized instructions used in the tablet-based MMSE-K administration

Domain	Item	Standardized Instruction (English)	표준 지시문 (Korean)
Orientation	Date	Please tell me today's date.	오늘 날짜를 말씀해 주세요.
Orientation	Place	Please tell me where you are now.	지금 계신 장소를 말씀해 주세요.
Registration (Memory)	3-word recall	I will say three words. Please repeat them after me: Apple, Table, Penny.	제가 세 단어를 말씀드리겠습니다. 따라 해주세요: 사과, 책상, 동전.
Attention & Calculation	Serial 7s	Please subtract 7 from 100, and keep subtracting 7 from each new number until I tell you to stop.	100 에서 7 을 빼고, 그 다음에도 계속 7 을 빼 주세요. 제가 멈추라고 할 때까지 하시면 됩니다.
Language	Naming	What is this called? (show picture of pencil/watch)	이것을 뭐라고 부르나요? (연필/시계 그림 제시)
Language	Repetition	Please repeat this sentence exactly as I say: "No ifs, ands, or buts."	제가 하는 말을 그대로 따라 해주세요: "아니면, 그러나, 또한 없이."
Comprehension	3-step command	Take this paper in your right hand, fold it in half, and put it on the floor.	이 종이를 오른손으로 잡고, 반으로 접은 다음, 바닥에 내려놓으세요.
Reading	Read & obey	(Show: "Close your eyes.") Please read this	(제시: "눈을 감으세요.") 읽고

		and do what it says.	지시에 따라 주세요.
Writing	Write sentence	Please write a complete sentence.	완전한 문장을 하나 써 주세요.
Drawing	Copy design	Please copy this picture. (Show interlocking pentagons)	이 그림을 똑같이 그려 주세요. (겹쳐진 오각형 제시)
