# Supplementary material for: Intra- and Inter-Rater Reliability Analysis of MMSE-K and Tablet PC-Based MMSE-K Kit in Patients with Neurologic Disease
Source: Healthcare (Basel). 2025 Nov 21;13(23):3015. doi: 10.3390/healthcare13233015 (PMC12692307; doi:10.3390/healthcare13233015)
Supplement: Supplementary file 1 [file healthcare-13-03015-s001.zip › Supplementary Table S-SUB1.pdf]

Supplementary Table S-SUB1. Descriptive subgroup results for tablet-based MMSE-K

Characteristics		*n*	%
**Sex**	Male	19	59.4
	Female	13	40.6
Age (year)	10s	1	3.1
	20s	0	0
	30s	1	3.1
	40s	1	3.1
	50s	5	15.6
	60s	6	18.7
	70s	17	53.1
	80s	1	3.1
Diagnosis	Thoracic myelopathy	2	6.2
	Intracerebral hemorrhage	4	12.5
	SAH	1	3.1
	Autoimmune encephalitis	1	3.1
	SDH	1	3.1
	Traumatic subarachnoid hemorrhage	1	3.1
	Cerebral infarction	8	25
	Secondary malignant neoplasm of the brain	1	3.1
	ICH	4	12.5
	IVH	1	3.1
	Subdural hemorrhage	2	6.2
	Neoplasm of spinal cord	1	3.1
	Meningioma	1	3.1
	Prostate cancer	1	3.1
	Neuromyelitis optica	1	3.1
	Giloblastoma of brain	1	3.1
